# An Investigation into the Viability of Portable Proximal Sensor X-Ray Fluorescence Data for Assessing Heavy Metal Contamination in Urban Soils: A Case Study in Changchun, China

**DOI:** 10.3390/toxics12110798

**Published:** 2024-10-31

**Authors:** Xiaoxiao Zou, Jilong Lu, Xinyun Zhao, Qiaoqiao Wei, Zhiyi Gou, Yaru Hou, Yawen Lai

**Affiliations:** College of Geo-Exploration Science and Technology, Jilin University, Changchun 130026, China; zouxx22@mails.jlu.edu.cn (X.Z.); zhaoxinyun@jlu.edu.cn (X.Z.); weiqiaoqiao@jlu.edu.cn (Q.W.); gouzy22@mails.jlu.edu.cn (Z.G.); houyr20@mails.jlu.edu.cn (Y.H.); laiyw@jlu.edu.cn (Y.L.)

**Keywords:** portable instruments, correction quality, soil investigation of quality, pollution index, Leopold Matrix

## Abstract

In order to validate the applicability of pXRF for rapid in situ detection of heavy metals in urban soils and to accurately obtain an assessment of soil quality in Changchun, a city in northeast China, 164 soil samples from within the main urban area of Changchun were collected for pXRF analysis. The main stable elements Si and Ti were used to establish a matrix effect correction model, and the values of Cr (64.2 mg⋅kg^−1^), Cu (43.8 mg⋅kg^−1^), Zn (96.2 mg⋅kg^−1^), As (20.9 mg⋅kg^−1^), and Pb (57.4 mg⋅kg^−1^) were predicted. The empirical findings indicate that the quality of soil data from the pXRF was improved to different degrees under the correction model, and it became a relatively reliable dataset; the order of improvement was Cu > Pb > Cr > Zn > As. A comprehensive assessment indicated that Changchun City is primarily contaminated by the heavy metals As, Pb, and Cu, with the main sources being automobile manufacturing and pharmaceutical chemical production. These findings align with previous studies and have produced favorable outcomes in practical applications. This rapid, non-destructive and economical detection method is very applicable and economical for the sustainable monitoring and control of heavy metals in large cities. This study provides a basis for rapid large-scale prediction of urban soil safety and protection of local human health.

## 1. Introduction

Urban soil is an organic component of the urban ecological system and the part of the pedosphere most affected by human activities. Large urban populations and community activities result in heavy metal contaminants entering the ground from various sources [1]. These sources include industrial activities, the use of wastewater for irrigation, vehicle emissions, and the extraction of metallic mineral resources [1,2,3]. In addition to these sources, the heavy metal content in urban areas is also influenced by background sedimentation and soil-forming matrices [4]. Soil organic matter exhibits a strong capacity for adsorbing heavy metals [5]. In general, heavy metal concentrations are higher in roadside soils compared to urban parks, and in urban areas compared to agricultural soils of the same parent material in remote suburban regions. Numerous studies have indicated that Cu and Zn levels are particularly significant in residential and mixed-use areas, while Pb and Cd levels tend to be elevated near industries and businesses associated with heavy metals [6]. Various human activities, such as heating and waste disposal, exert differing impacts on the concentrations of these heavy metals. Solid particulate matter from industrial activities enters the atmosphere, where it can be deposited and subsequently migrate to water bodies through rainfall runoff [7]. This process facilitates the accumulation of heavy metals in urban environments. The limited capacity of urban soils, combined with the slow rate of internal material cycling, further impedes the degradation of heavy metals, thereby posing significant risks to human health. Exposure can occur through oral ingestion, skin contact, and inhalation, leading to a range of health issues. For instance, As and Cr are highly carcinogenic, while Pb, Cu, and Zn are associated with blood disorders and nervous system diseases [8]. This presents a significant risk to the well-being and existence of the urban community. Given its central role in the population and that it is closely related to human survival and health, it is crucial to promptly detect and address soil contamination in urban regions [9,10].

However, traditional laboratory analyses, such as Inductively Coupled Plasma Mass Spectrometry (ICP-MS), have long detection times, complicated operations, and require experimental consumables such as acid reagents [3]. This not only incurs high economic costs but also easily leads to further pollution. Portable energy dispersive X-ray fluorescence (pXRF) instruments are known for their rapid detection and low-cost, non-destructive analysis, making them suitable for in situ heavy metal detection in the field and real-time acquisition of soil geochemical data [11,12,13,14]. The accuracy of pXRF analysis is limited by matrix effects that are inherent and cannot be completely eradicated, making it challenging to directly compare its accuracy to that of conventional laboratory analysis techniques [15,16]. By increasing the sample size and implementing rigorous calibration methods, the precision of pXRF analysis technology can be enhanced to fulfil the requirements of large-scale urban pollution assessment [17]. Currently, there are two main types of calibration method: one involves experimental operations, such as the standard built-in method and dilution method for suppression, while the other utilizes mathematical modelling, including linear regression and neural network methods [18]. The practical implementation of the experimental method is complex and time-consuming, especially when working with a large number of samples. Accordingly, a new matrix effect correction mathematical model method for calibration has been developed, which is more suitable and convenient for the timely implementation of experimental decisions, such as the design of an encrypted sampling grid and the conducting of repeated experiments. 

PXRF technology has significant potential for application in alloy analysis, archaeological research, and mining ore detection [19]. Although urban soils have been studied by experts in the past, they tend to be mostly in suburban areas and forested farmland near cities, etc., and there are relatively few examples of rapid contamination monitoring using pXRF in medium- to large-scale major built-up areas of cities [20]. In the last five decades, Changchun, an important city in northeast China in terms of its urbanized area, has experienced urbanization fueled by industrial development [21]. Many factories have been established that may discharge a large number of heavy metals, among which the main pollutants associated with urbanization are chromium (Cr), copper (Cu), zinc (Zn), arsenic (As), and lead (Pb) in Changchun, and these five metals pollute the soil more commonly than other heavy metals [22,23]. We hypothesized that pXRF data after correction could accurately represent the heavy metal content in the soil, with an accuracy comparable to that of traditional elemental measurements, thereby meeting the standards for quantitative analysis. Changchun, a typical industrialized city in China, is affected by various urban activities, which may lead to heavy metal concentrations in the soil that exceed safety standards and potentially jeopardize the health of residents. Therefore, timely monitoring to detect contamination is essential. The use of pXRF effectively addresses these monitoring needs, making it an appropriate solution for this purpose. To test this hypothesis, we applied pXRF in situ analysis, predicted the estimates under the constructed correction model, and used multiple evaluation factors to judge the accumulation level of elements. Additionally, the research provides a reference for analyzing other heavy metal elements and includes technical details on implementing this method in fieldwork. This was conducted with the objective of ensuring the safety and health of the local population. In light of the aforementioned findings, recommendations have been formulated pertaining to this matter. Therefore, the objectives of this paper are 1. to assess the applicability of pXRF for analyzing heavy metal content in urban soils; and 2. to evaluate the soil quality of Changchun City based on the precise data obtained.

## 2. Materials and Methods

### 2.1. Overview of the Study Area

**Figure 1:**
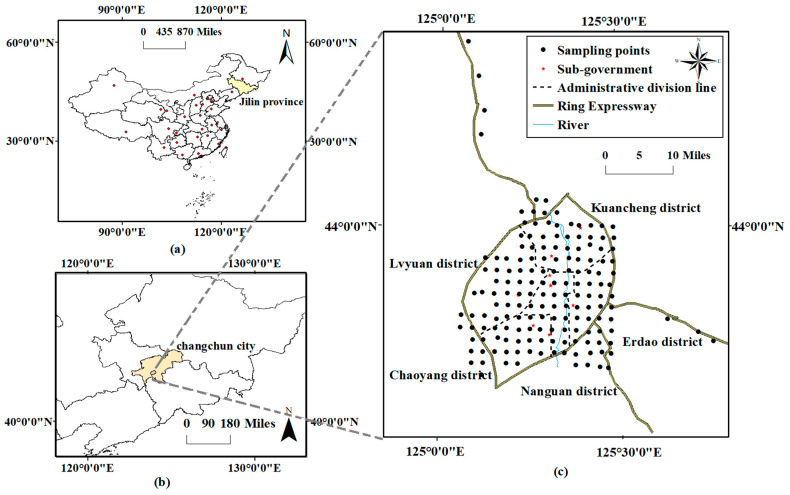
Map of the study area and distribution of soil sampling points. (**a**): Map of Administrative Areas in China, (**b**): Map of Administrative Areas in Jilin Province, (**c**): Changchun main urban area map.

This research was conducted in the urban area of Changchun (124°18′–127°02′ E, 43°05′–45°15′ N), which is the capital city of Jilin province, located in the hinterland of the Songnen Plain and which serves as the geographical core of northeast China (Figure 1) [24]. Changchun has a temperate continental semi-humid climate, with an average annual temperature of 4.8 °C, annual precipitation ranging from 600–700 mm, and a freeze period lasting for 5 months annually [25,26]. According to the WRB standards and the China Soil Database, the main inherent soil types in Changchun are black soils and meadow black soils. According to the National Standard of the People’s Republic of China (HJ962-2018), the potentiometric method is mainly used for the determination of soil pH, and the water–soil ratio is 2.5:1, while the method for the determination of soil organic matter is mainly the potassium dichromate oxidation method [27,28,29]. The soil type in the eastern part of Changchun City is alfisol, primarily characterized by a clay loam texture, a pH level generally around 7.0, and low organic matter content. In the central area of Changchun, the soil is phaeozem, which has a loamy clay texture, with a pH range of 7.1 to 7.5 and an organic matter content exceeding 2.0%. In the western region, the soil type is Chernozem, featuring an organic matter content greater than 2.0% in the topsoil. This soil typically exhibits weak alkalinity, with a topsoil pH of approximately 7.0, and has a texture of either loamy clay or sandy clay. Before the development of the urban area, the predominant soil types included black soil, meadow soil, and meadow black soil. The soil pH in the Changchun urban area ranged from 7.5 to 8.1, indicating a weakly alkaline condition. Additionally, the soil texture was primarily medium loamy, characterized by a deficiency in organic matter. The city covers an area of built districts of approximately 551.38 km^2^. It has a permanent population of 9,087,200, consisting of 3,575,300 urban inhabitants, with an urbanization rate of 66.8% in 2022. Changchun, recognized as the cradle of the automobile manufacturing industry, has produced and assembled a total of 1.56 million automobiles as of 2023. Additionally, it serves as a biomedical hub, boasting an industrial scale of 97.8 billion yuan and a comprehensive industrial chain encompassing medical devices and chemical pharmaceuticals [30]. The city boasts a comprehensive array of industrial facilities, including machinery manufacturing, biomedical equipment, chemical manufacturing, bus manufacturing, power and heat production, and other related sectors [31,32].

### 2.2. Sampling and Analysis

#### 2.2.1. Sampling and Analysis with Sensors (pXRF)

##### Quality Assurance and Quality Control (QA/QC)

To ensure the usability of the data for decision-making purposes, this study conducted tests on international standard samples to verify the accuracy and precision of the data. The international standard geochemical analysis sample number used for this analysis is the GSS for Soil Geochemical Standard. A total of 18 international standard geochemical samples were used in this study for quality control purposes (Table 1). In order to ensure temporal and spatial consistency in the data analyses, standards are analyzed concurrently with the soil samples. The calibration of these standards is conducted approximately every ten soil samples. The standard samples are placed in a slightly flattened cylindrical container, covered with a film, and subsequently tested using pXRF.

For the testing, a portable energy-dispersive X-ray fluorescence spectrometer (X-Met 7500) manufactured by the Oxford Instruments Group in the UK was used. The device operated at a voltage of 40 KV, a current of 60 mA, and possessed a maximum power output of 2.4 kW [13]. Additionally, the instrument was equipped with a fourth-generation Silicon Drift Detector (SDD) and a Rhodium (Rh) anode target [33,34]. It also offered the option to select the soil test modes Soil-Mining_fp and Mining_LE_FP, which were based on the basic parameter method and empirical coefficient method (they are usually inbuilt algorithms carried out by the manufacturer). The standard sample was placed in the pXRF scan 5 times; each time the analysis duration was 60 s, and the final average value was obtained as the pXRF analysis result. We performed a simple linear fit using the standard values of the standard samples (quality guaranteed by the quality analysis of the national production unit) and the results of the pXRF test and found that the fit was in accordance with the test (R^2^ > 0.95). The results of the analyses of the international standard geochemical samples are shown in Table 1 below. The calculated R^2^ values for the elements Cr, Cu, Zn, As, and Pb are 0.963, 0.978, 0.992, 0.999, and 0.999, respectively. The recovery rate is 100%, except Pb, which is also 62%. This is considered an acceptable level of reporting rate for general research [33]. This quality control and assurance demonstrate that the pXRF data from this study are credible and comparable to studies by other researchers in our experimental group for both conventional laboratory XRF and the pXRF used in the experiments [35]. The detection limit and RSD of portable XRF is Cu (10 mg⋅kg^−1^, 6.3%), Cr (10 mg⋅kg^−1^, 8.6%), Zn (5 mg⋅kg^−1^, 15.4%), As (4 mg⋅kg^−1^, 12.5%), and Pb (5 mg⋅kg^−1^, 7.4%). 

##### Analysis of Soil Samples

During the sample collection process, sampling points were systematically distributed based on a grid pattern, with each point spaced at 2 km intervals. Surface soil within the study area was excavated, and soil samples were obtained at a depth of 0–20 cm using a stainless-steel spade. Additionally, soil specimens were gathered from 3 to 5 different locations surrounding each sampling point, combined, and thoroughly mixed to create a composite sample after the removal of any extraneous materials such as branches, twigs, and leaves. A total of 164 samples were acquired for this study, consisting of 156 samples from the primary urban zone and 8 samples as the background soil from Ring Express (Figure 1c).

For the pXRF test, after removing stones, wood splinters, or some extraneous matter, placing it in a cloth bag and tapping it evenly with a wooden stick, and air-drying it in the sun for about ten minutes, then the air-dried sample was placed into a short cylindrical plastic cup with a diameter of 50 mm and a depth of 20 mm and flattened evenly with paper to facilitate scanning [36]. The experiment was replicated five times at distinct and uniformly distributed locations within a sample. Each test lasted for 60 s, resulting in a total of 600 s for one sample measurement. Finally, the mean value of the five times was calculated as the final result. Based on previous data-processing experience, we filled in the missing data for undetected samples with half the minimum value of the test [37]. To obtain the necessary data, we stored the remaining soil samples and sent them to a laboratory for conventional testing, which included ICP-MS test methods. The study measured the Cr, Cu, Zn, As, and Pb contents using pXRF, in addition to the major elements selected based on element correlation analysis, which included Si and Ti. The experimental data satisfy the precision criteria for data adequacy. The findings from the study of variance (ANOVA) revealed a statistically significant disparity (*p* < 0.05) between the in situ portable XRF measurements and the conventional laboratory data obtained (ICP-MS).

#### 2.2.2. Data Processing and Methods

##### Calculation of the Correction for Matrix Effects

In theory, the matrix effect arises from various factors. In the context of chemical analysis, the matrix effect refers to the influence on an analytical method caused by all other components of the sample except the specific compound being quantified; the content of an element is affected by all elements except the element being measured [38]. In the actual calculation process, due to the problems of calculation workload and accuracy, it is complicated to calculate all elements one by one. Simplification of the original Sherman equation can help minimize spectral noise and address spectral interferences when analyzing the multi-variable XRF spectra (Equation (1)).

According to the stability tested by the instrument, the main elements such as Si and Ti could be selected as the primary correction indicators in this paper. They have a high content, are minimally influenced by other elements, and are relatively stable in the crust. Multivariate linear matrix effect correction was then carried out on the remaining elements to be measured. On one hand, the impact of the outlier on the correction results diminished compared to the traditional linear regression method (LR); on the other hand, the calculated quantity was simplified.

(1) $$C_{i}^{\prime }=\alpha_{i}C_{i}+\beta_{j}Si+\gamma_{z}Ti+\mu_{i}\;$$

where $C_{i}^{\prime }$ is the corrected content of heavy metals, (element i) is the predicted value, $C_{i}$ is the content of elements established by pXRF, Si and Ti is the test content established by pXRF, $\alpha_{i}$ is the regression coefficient of heavy metal element i, $\beta_{j}$ and $\gamma_{z}$ are the influence coefficients of the major element Si and element Ti, and $\mu_{i}$ is the regression intercept of heavy metal element i (Equations (2)–(5)) [39,40], all of which are employed by multivariate partial least squares regression and multiple linear regression.

(2) $$M_{i}=C_{i}-\sum_{k}\frac{C_{\mathrm{ik}}}{k}$$

(3) $$\alpha_{i}=\frac{\left(\sum_{k}{M\prime }_{\mathrm{ik}}M_{\mathrm{ik}})\left(\sum_{k}M_{\mathrm{jk}}^{2}\right)-(\sum_{k}{M\prime }_{\mathrm{ik}}M_{\mathrm{jk}})(\sum_{k}M_{\mathrm{ik}}M_{\mathrm{jk}}\right)}{(\sum_{k}C_{\mathrm{ik}}^{2})(\sum_{k}C_{\mathrm{jk}})-{\left(\sum_{k}C_{\mathrm{ik}}C_{\mathrm{jk}}\right)}^{2}}$$

(4) $$\beta_{j}=\frac{\left(\sum_{k}{\;M\;\prime }_{\mathrm{ik}}M_{\mathrm{jk}}\right)(\sum_{k}M_{\mathrm{ik}}^{2})-(\sum_{k}{M\prime }_{\mathrm{ik}}M_{\mathrm{ik}})(\sum_{k}M_{\mathrm{ik}}M_{\mathrm{jk}})}{(\sum_{k}C_{\mathrm{ik}}^{2})(\sum_{k}C_{\mathrm{jk}}^{2})-{\left(\sum_{k}C_{\mathrm{ik}}C_{\mathrm{jk}}\right)}^{2}}$$

(5) $$\mu_{i}=\frac{\left(\sum_{k}{C\prime }_{\mathrm{ik}})-\alpha_{i}(\sum_{k}C_{\mathrm{ik}})-\beta_{j}(\sum_{k}C_{\mathrm{jk}}\right)}{k}$$

where n is the number of samples and C_ik_ and C_jk_ are the testing values of elements i and j in sample k, respectively [41]. The testing values of i and j are zero-centered to M_ik_ and M_jk_ (Equations (6) and (7)) [42]. Correlation coefficients can be calculated by testing standard samples before testing due to instrument commissioning. Given the complexity of these calculations, this study employs software (such as IBM SPSS Statistics 21.0) that provides such analyses through a streamlined one-step process, as opposed to manual calculations. This approach not only alleviates computational challenges but also enhances accuracy.

(6) $$M_{\mathrm{ik}}=C_{\mathrm{ik}}-\frac{\sum_{k=1}^{n}C_{\mathrm{ik}}}{k}$$

(7) $$M_{\mathrm{ij}}=C_{\mathrm{jk}}-\frac{\sum_{k=1}^{n}C_{\mathrm{jk}}}{k}$$

Performance statistics were used to evaluate the correction results, which included the coefficient of determination (R^2^), mean absolute error (MAE) and root mean squared error (RMSE), the ratio of performance to interquartile distance (RPIQ) (Formulas (8)–(11)). Both the RMSE and R^2^ metrics evaluate the performance of the linear regression model on the dataset. MAE and RMSE assess the regression model’s capability to predict the absolute value of the response variable. In addition, R^2^ assesses the predictor’s ability to elucidate the change in the response variable [43]. The relationship between the pXRF testing value and an element’s predicted value (corrected value) could be estimated using the R^2^ values. A stronger correlation is indicated by a larger R^2^ value. As R^2^ approaches 1, it indicates that the regression model is better fitted. In general, a higher RPIQ indicates a better predictive ability for the model.

(8) $$R^{2}=\frac{\sum_{N}(C_{m}-{\bar{C_{n})}}^{2}}{\sum_{N}(C_{n}-{\bar{C_{n})}}^{2}}$$

(9) $$\mathrm{RPIQ}=\frac{Q3-Q1}{\mathrm{RMSE}}$$

(10) $$\mathrm{RMSE}=\sqrt{\frac{\sum_{N}{\left(C_{m}-C_{N}\right)}^{2}}{N}}$$

(11) $$\mathrm{MAE}=\frac{\sum_{N}|C_{m}-C_{n}|}{N}$$

where N is the number of samples, $C_{m}$ is the predicted values, $C_{n}$ is the ICP-MS value (represented true values), $\bar{C_{n}}$ is the mean value of $C_{n}$, Q3 is the third quartile, and Q1 is the first quartile distance.

##### Pollution Evaluation Methods

The overall concentration of heavy metals and the statistical methods do not provide comprehensive information on the extent of soil contamination. They only give a rough indication of the potential for contamination [44]. The pollution index can be used as a tool for comprehensive geochemical assessment of soil environmental status [45]. Single contamination indices may occasionally overestimate the level of contamination at a site due to the methodologies employed in calculations and the selection of background values. Conversely, more aggregated contamination factors may be influenced by varying choices of contaminant element types or may neglect the impact of natural geochemical variability, resulting in inaccurate evaluation outcomes. Therefore, this study employs a diverse array of pollution indicators, encompassing both traditional metrics such as single pollution factors and more comprehensive methodologies for pollution assessment [46] (Table 2). The geochemical background values used in this paper are the soil geochemical background of Jilin province (Table 3). Pollution evaluation is essential for understanding the extent of pollution. This paper employs absolute principal component analysis–multiple linear regression to identify the contributions of various pollution sources [47]. The analysis was primarily conducted using relevant software for calculations.

##### Environmental Impacts

Determining the sources of pollution is essential for conducting a thorough and accurate environmental impact assessment [56]. The impact assessment tool utilized in this study is the Leopold Matrix. This is a two-dimensional interactive matrix, where the horizontal axis represents the environmental factors affected by pollution, including the physical environment and the social environment, among others [8]. The vertical axis denotes the environmental impact factors, which primarily consist of activities that may cause pollution, such as industrial processes and human activities [57]. To present the results more effectively, we utilized a fractional representation (M/I), where M denotes the magnitude of the change in the impacting activity (with values ranging from −5 to +5; positive values indicate positive impacts, while negative values signify negative impacts) and I represents the importance of the change to the environment (on a scale from 1 to 10) [58]. Ultimately, the environmental impact is quantified as the product of M and I, resulting in the environmental impact score [59].

## 3. Results and Discussion

### 3.1. Evaluation of Matrix Effect Correction 

**Figure 2:**
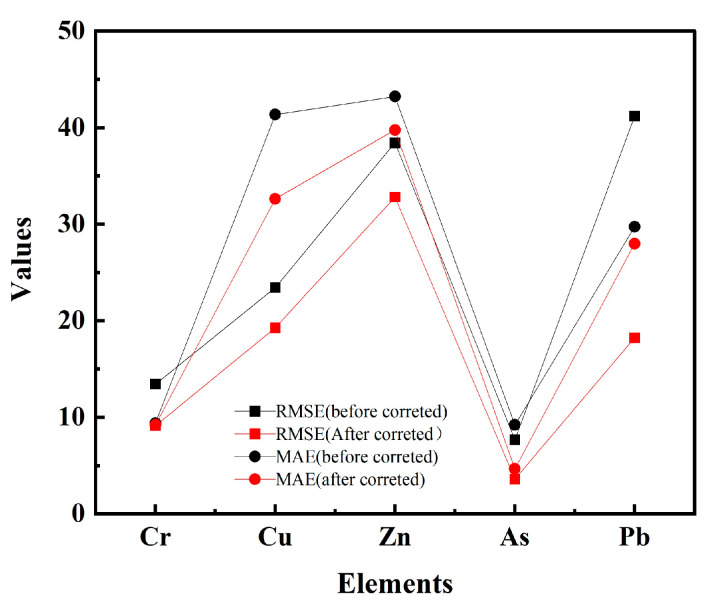
The MAE and RMSE values for the correction results of the improved methods.

**Figure 3:**
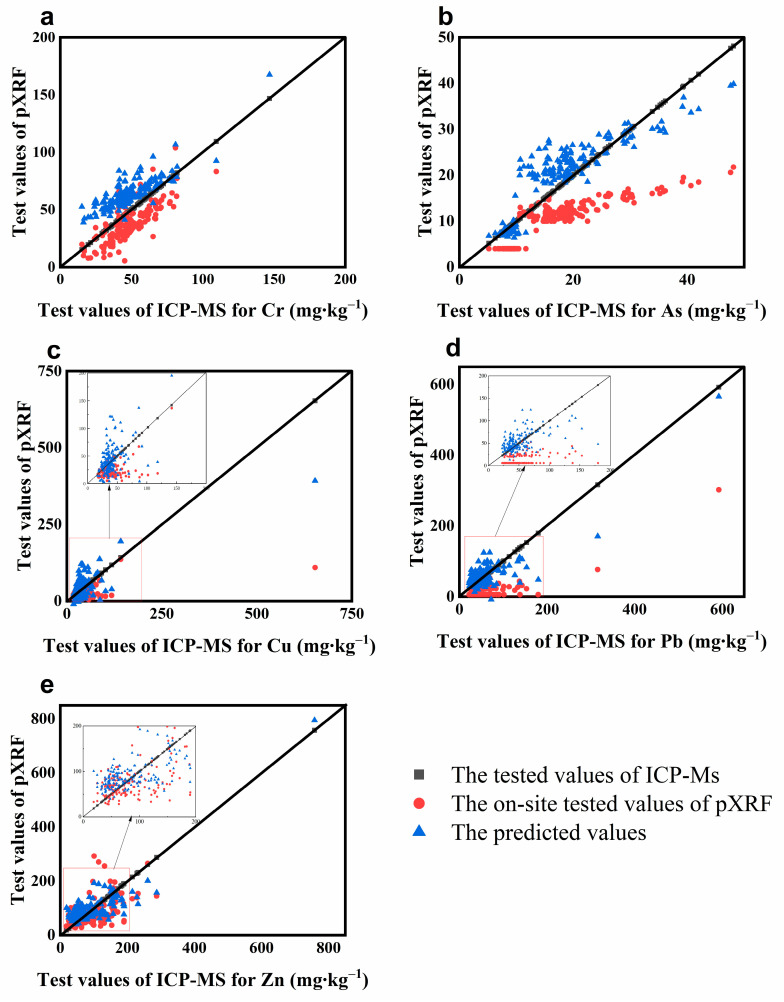
Comparison of scatter plots before and after correction for heavy metal elements (**a**–**e**).

The Si–Ti matrix effect correction method and the traditional linear regression (LR) method were used to correct the test results and fit the image linearly. A black diagonal line going through the origin is a 1:1 line as we assumed that the ICP-MS data represented the true values (Table 4 and Table 5 and Figure 2 and Figure 3). The mean concentrations of five element concentrations determined by in situ pXRF which were relatively lower than data obtained by ICP-MS data. It is noteworthy that the mean of the in situ Zn pXRF (90.1 mg⋅kg^−1^) is very close to the mean of the ICP-MS (91.12 mg⋅kg^−1^) analyses. These results show that pXRF underestimates soil metal concentrations. Validation indices of MAE and RMSE are shown in Figure 2. The traditional LR generated a lower correction quality than the Si–Ti matrix effect correction method. Although the values for MAE and RMSE remain high within reasonable limits, a significant improvement is evident in the corrected data, which may be due to outliers and model fitness [60].

To better demonstrate the applicability of the model, we also performed validation. The RPIQ values of the element Cr (RPIQ = 1.572) and As (RPIQ = 1.168) are all greater than 1, showing excellent accuracy reliability, and the RPIQs of Cu (RPIQ = 0.968) and Pb (RPIQ = 0.828) are both greater than 0.8, with high accuracy, but the RPIQ of Zn (RPIQ = 0.792) is less than 0.8 but greater than 0.7, which is still in the acceptable range. The above shows that the calibration results for the five heavy metal elements are reasonable and reliable.

We can see that the corrected data for the elements Cu and Pb are better fitted to the true values (ICP-MS test values) than the uncorrected ones. Element Zn tests higher than the true value at data points with high content, but elements Cu and Zn test lower than the true values. The corrections are closer to the actual values for the high-value ranges of elemental As, but for elemental Cr, the predictions are slightly larger than the true values, but still within reasonable limits, which may be related to the instrumental detection limits (Figure 3).

Compared to traditional and linear regression corrections, the R^2^ values for the Cr, Cu, Pb, and Zn elements significantly increased. Cu showed the most significant improvement, followed by Pb, Cr, Zn, and finally As. The R^2^ values were greater than 0.75 after calibration, indicating a strong correlation with laboratory testing values obtained through ICP-MS. These corrections have improved the data quality to some extent and weakened the influence of the matrix effect. The comprehensive comparison of elements should be ranked as Cu > Pb > Cr > Zn > As (Table 4). This ranking could be attributed to the constituents’ amalgamation or their intrinsic characteristics.

### 3.2. Assessment of Pollution Degree

**Figure 4:**
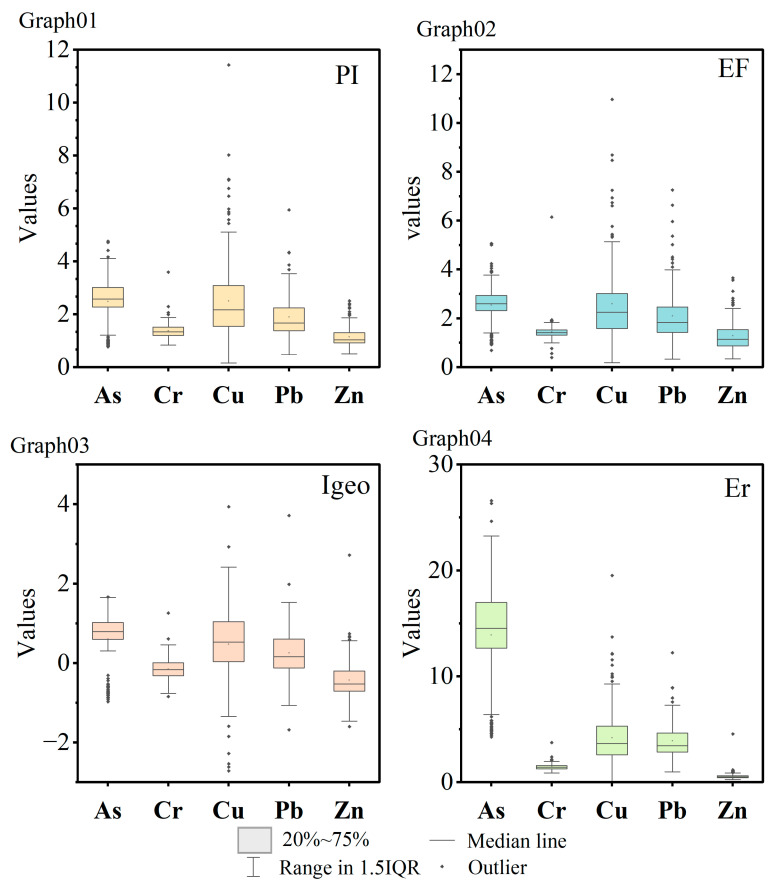
Box plot of pollution index distribution (Graphs 01, 02, 03, 04 correspond to pollution indices PI, EF, Igeo, Er, respectively).

The pollution index statistics for the built-up area of Changchun City are shown in Figure 4 below. These box plots illustrate the distribution of the final calculated values for each evaluation method. Additionally, various levels of contamination can be inferred based on the different ranges of contamination value divisions. In the box plot illustrating the distribution of the PI calculation results (Figure 4—Graph 01), the majority of samples exhibit values below three, indicating slight to moderate contamination. However, a few sample points demonstrate severe contamination. In the calculations of the enrichment factor index (Figure 4—Graph 02), most distributions are below five, and none exceed twenty. This indicates a slight to moderate enrichment of the element. Similarly, in the ground accumulation index distribution (Figure 4—Graph 03), most of the samples are in the range of 1–2, showing slight cumulative contamination, and in the final distribution of the ecological risk factor, the data show that most of them are also below 20, but As has a higher ecological risk compared to the other elements (Figure 4—Graph 04). However, considering the toxicity of As and the background level of soils in Jilin province, this could be linked to the selection of background values for the element, which may amplify its contamination effects. The average value of the Nemerow index is equal to 1.20, indicating that the soil standards in the built-up area of Changchun are mildly polluted (Table 6). When calculating the PLI, it can be observed that As has the greatest contribution among the five elements, followed by Cu, Pb, Cr, and Zn. 

**Figure 5:**
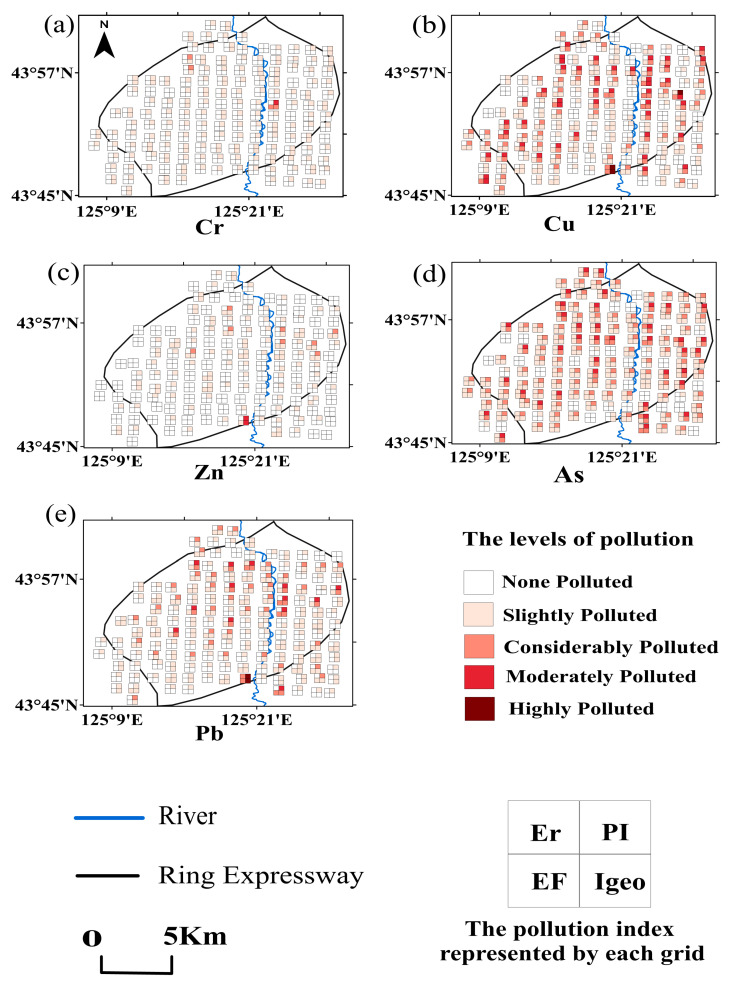
Spatial distribution of point pollution in Changchun City based on four pollution assessment methods. The subplot represents the degree of heavy metal contamination at each sampling site in the main urban area of Changchun, (**a**) the pollution index level of element Cr; (**b**) the pollution index level of element Cu; (**c**) the pollution index level of element Zn; (**d**): the pollution index level of element As; (**e**): the pollution index level of element Pb.

Figure 5 illustrates the spatial distribution of heavy metal pollution in the built-up area of Changchun. Each small square in the figure represents the evaluation value of a specific pollution assessment method, with the degree of pollution indicated by a gradient of red colors. Four squares together form a larger square that represents the pollution level at a given sampling point. The greater the number of red squares at a sampling point, the higher the degree of pollution [61]. The distribution of significant contamination for the elements lead, copper, and zinc are similar in the southeast corner, with contamination in this area showing large areas of red color, representing significant contamination regardless of the method of calculation, while the distribution of elemental copper in the northwest corner changes. The combination of several evaluation methods indicates that the level of Cr contamination was low and that the background values of Cr in the soil were not significantly elevated compared to the test values. However, it is important to note that Cr is relatively toxic and requires careful monitoring even at low levels of contamination [62]. The evaluations conducted using various methods indicate differing levels of pollution, which must be assessed carefully and comprehensively. Notably, most heavily polluted sample sites are concentrated around residential areas and industrial parks.

**Figure 6:**
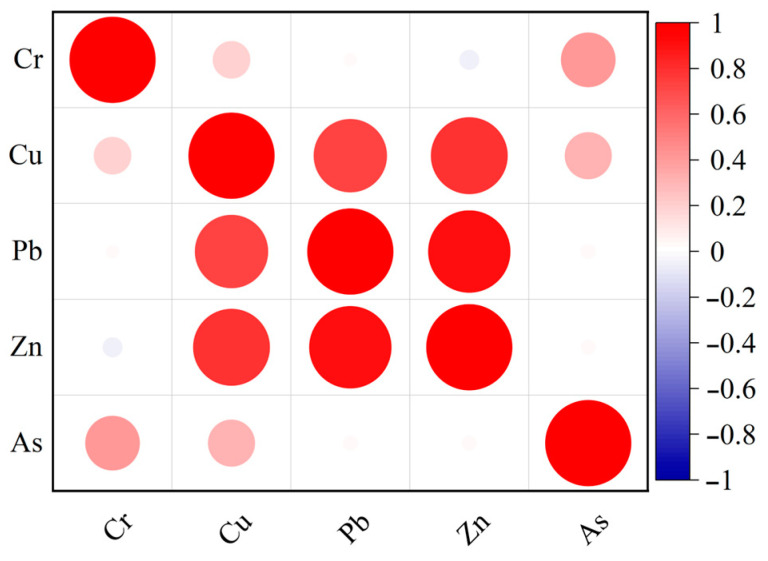
Correlation analysis of heavy metals in soil in the main urban area of Changchun City.

The correlation heat map of heavy metal concentrations in Changchun soil indicates that, at a significance level of *p* = 0.05, the concentrations of Cu, Pb, and Zn are highly correlated, with correlation coefficients exceeding 0.8 [63]. In contrast, the elements of As and Cr show a weak correlation, approximately 0.5 (Figure 6). Combined with the contour plots of these elements, it is evident that there may be homology between these subgroups [64]. Principal component analysis similarly showed that a total of two principal components emerged between the five elements [65]. By employing Kaiser’s standard orthogonal rotation method, the eigenvalues of the two factors were determined to be 2.68 and 1.44, respectively [66]. Analyzing the coefficients of the rotated component matrices revealed that Source 1 exhibited higher loadings for Cu, Pb, and Zn, while Source 2 showed greater loadings for As and Cr. These findings were consistent with the results of the correlation analyses (Table 7). The cumulative variance contribution of the principal components reached 82.37%, with the first two principal components explaining 53.59% and 28.77% of the total variance, respectively [67,68]. An absolute principal component multiple linear regression analysis was conducted, revealing that all R-squared values exceeded 0.65, indicating the model’s applicability. The identified pollution sources comprised two known sources and one unidentified source.

**Figure 7:**
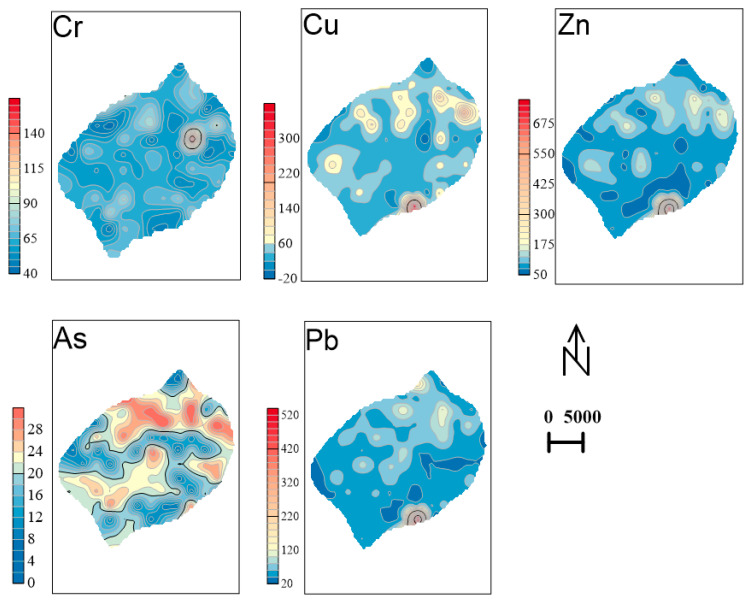
Spatial distribution of elements (Cu, Pb, Zn, As, Cr) in the main urban area of Changchun City.

Source 1 primarily contributes to the levels of Pb and Zn, while Source 2 has a greater impact on As and Cr. Additionally, the unknown sources show some correlation with Cu, Pb, Zn, and Cr. Notably, Cu is significantly associated with all three sources of pollution. We scrutinized the descriptive records at the time of sampling, as well as the views of satellite maps and the site. The contour map indicates that high concentrations of Cu, Pb, and Zn are present in the lower reaches of the river (Figure 7). Our sampling records reveal multiple sewage pipes in these areas, as well as metal product manufacturing facilities that discharge wastewater, including from electroplating processes, into the watershed. This has resulted in soil pollution in the surrounding regions [22]. Notably, these contaminated sites overlap with several transportation routes, such as Line 2 of the underground system and railway stations. Previous studies have demonstrated that traffic contributes to the accumulation of Pb and Zn content in the soil [23]. Therefore, the primary source of pollution can be attributed to metal product manufacturing activities. Source 2 primarily contributes to the levels of As and Cr. In the spatial distribution map of As, this coincides with numerous residential areas. In the vicinity of the high-concentration zones, our records reveal the presence of several abandoned factories [5]. Changchun City experiences severe winters, often relying on coal or electric heating. The combustion of coal releases atmospheric soot that contains heavy metals such as As, Cr, Pb, etc., which accumulate in the soil through atmospheric deposition, resulting in a broad area of impact [27]. Therefore, Source 2 is predominantly a coal-fired heating source. Similarly, the unidentified Source 3 contributes to the levels of a comprehensive assessment suggesting that these elements are primarily associated with the chemical manufacturing industry [65]. The production of chemical raw materials or products generates complex chemical products, which not only produce wastewater but also result in significant emissions and residual waste [32]. Consequently, this unidentified source is mainly for chemical pollution.

### 3.3. Assessment of Leopold Matrix 

The Leopold Matrix was evaluated mainly by several environmental experts associated with Changchun City (Table 8). In the Leopold Matrix, our list of environmental activities with the highest impact on the soil are the waste recycling activities of the automotive industry and the manufacturing of pharmaceutical and chemical products, with negative impact evaluation scores of twelve and nine, respectively. The chemical–pharmaceutical industry has the highest score (20.10) for each environmental factor, representing a significant negative impact. The average value of the impact on soil for each activity is 2.67 less than 3.0, which indicates that the activities are all still within the acceptable range. The impact value for all activities is 32.04 for soil, and the total impact values for water and air are 27.88 and 28.36, respectively. Activity factors such as automobile manufacturing and chemical–pharmaceutical industries currently have a relatively large negative impact on the environment, but all are still within the alert range and require treatment activities.

## 4. Conclusions

The conclusions we can draw are as follows:

1. The Changchun built-up area as a whole is slightly to moderately polluted, but it needs to be alerted to the contamination of elemental As, as well as Cu and Pb, with the main sources of pollution being metal-related industrial manufacturing, the manufacturing of chemical products, and coal-fired heating. The environmental impacts of activities in the urban areas of Changchun are all within manageable limits and soil remediation should be carried out for the corresponding response sites immediately.
2. The on-site test data obtained by pXRF can be considered as a reliable dataset after processing by the correction model. The order of correction for each element under this simple correction model is as follows: Cu > Pb > Cr > Zn > As. This exploratory correction method can be extended to the correction of other elements, which also provides a valuable reference for the correction of in situ measurements of other potential soil pollutants.
3. The pXRF is efficiently calibrated for real-time scanning of regional soil contamination and large-scale sustainable rapid assessment. Therefore, we advocate that calibrated pXRF data from proximal sensors can be used by government agencies or monitoring organizations as complementary information to enhance spatial monitoring of potentially contaminated sites at the local and regional levels to ensure the safety and health of populations in urban environments.

## Figures and Tables

**Table 1:** Results of certified values of national standard soil geochemical samples (St) and test values of pXRF (pXRF).

	Cr (pXRF)	Cr (St)	Cu (pXRF)	Cu (St)	Zn (pXRF)	Zn (St)	As (pXRF)	As (St)	Pb (pXRF)	Pb (St)	Si % (pXRF)	Si (St)	Ti (pXRF)	Ti% (St)
GSS-1a	51.2	44.00	55.0	42.00	677.0	475.00	48.6	33.00	421.8	339.00	30.62	26.46	3956.4	0.326
GSS-1	45.0	47.00	21.60	21	863.8	680	44.4	34	102.6	98	30.47	29.263	5885.2	0.483
GSS-2	370.8	410.00	30.0	16.30	45.2	42.00	16.6	13.70	-	20.00	33.87	34.29	2954.2	0.271
GSS-3	70.8	70.00	14.00	11.4	19.6	31	-	4.4	20.0	26	34.88	34.929	2378.2	0.224
GSS-4	31.6	25.00	18.00	40	214.4	210	73.0	58	62.8	58	23.42	23.817	13,798.2	1.080
GSS-5	36.8	43.00	118.0	144	605.4	494	530.8	412	690.6	552	24.04	24.575	7633.4	0.629
GSS-6	50.4	57.00	478.60	390	101.2	97	302.2	220	371.0	314	25.09	26.613	5556.	0.439
GSS-7	63.4	82.00	91.4	97.00	149.6	142.00	-	4.80	18.8	14.00	16.25	15.28	25,629.0	2.020
GSS-8	61.0	62.00	18.00	24.3	60.4	68	17.0	12.7	-	21	28.65	27.398	4510.20	0.380
GSS-14	51.2	66.00	29.0	27.40	109.4	96.00	-	6.50	32.6	31.00	30.28	30.16	4700.0	0.406
GSS-17	45.0	61.00	20.4	12.60	28.2	29.00	11.6	6.20	-	17.40	36.17	36.60	1874.0	0.191
GSS-20	80.0	92.00	25.4	28.00	54.6	61.00	11.2	8.70	-	13.40	24.09	22.10	3392.6	0.330
GSS-22	62.4	62	19.8	18.30	65.6	59.00	13.8	7.80	25.0	26.00	32.61	31.90	4395.8	0.380
GSS-23	28.20	32	40.4	32.00	122.4	97.00	20.8	11.80	-	28.00	29.954	27.95	5946.4	0.500
GSS-24	351.40	370	31.6	28.00	96.2	81.00	24.0	15.80	30.2	40.00	32.130	32.31	5361.4	0.450
GSS-25	166.40	118	24.0	23.60	75.8	66.00	17.6	12.90	-	22.00	30.894	28.48	4494.2	0.390
GSS-26	122.20	75	21.8	19.10	68.2	62.00	12.5	8.90	-	21.00	32.830	30.92	4539.0	0.410
GSS-27	64.60	68	52.8	54.00	153.4	127.00	17.7	13.30	44.0	41.00	29.324	27.52	7457.8	0.640

Notes: Unless otherwise indicated, units are in mg⋅kg^−1^.

**Table 2:** Pollution indices used and description of the corresponding parameters.

Index	Formula	Explain
Single pollution index (PI)	$\mathrm{PI}=C_{n}/B_{n}$	C_n_—the content of the heavy mental element [48]. B_n_—the geochemical background value of Jilin province.
Geo-accumulation index (I-_geo_)	$I_{\mathrm{geo}}=\mathrm{Log}\left(C_{n}/k\times B_{n}\right)$	C_n_—the measured levels of the heavy metal “n” in the soil sample, B_n_—used the same way as PI, and K is the correction factor, which was chosen as 1.5 [44].
Contamination factor (CF)	$\mathrm{CF}=C_{n}^{i}/C_{p}^{i}$	C_n_—the content of heavy metal from at least five samples of individual metals, C_p_—pre-industrial reference value for the substances [49].
Enrichment factor (EF)	$\mathrm{EF}=\left(C_{n}/C_{\mathrm{ref}}\right)/\left(B_{n}/B_{\mathrm{ref}}\right)$	C_n_—content of analyzed heavy metal, C_ref_—one of the following metals, Ti [50]. B_n_—reference content of the analyzed heavy metal, B_ref_—one of the following metals, Ti in the background [51].
The pollution load index (PLI)		CF_n_—the contamination factors of the element n [52].
Risk factor (RI)	$\mathrm{PLI}={\left({\mathrm{CF}}_{1}+{\mathrm{CF}}_{2}+\cdots +{\mathrm{CF}}_{n}\right)}^{\frac{1}{n}}$ $E_{r}^{i}=T_{r}^{i}\times C_{f}^{i}$ $\mathrm{RI}=\Sigma_{i=1}^{n}E_{r}^{i}$	n—the number of heavy metals, Tr—the toxicity response coefficient of an individual metal, C_f_—contamination factor, Er—single index of the ecological risk factor [53].
Degree of contamination (C-_deg_)	$C_{\deg}=\Sigma_{i=1}^{n}{\mathrm{CF}}_{i}$	CFi—the contamination factor for each element [54].
Nemerow pollution index (PI-_Nemerow_)	${\mathrm{PI}}_{\mathrm{Nemerow}}=\sqrt{\frac{{\left(\frac{1}{n}\Sigma_{i-1}^{n}\mathrm{PI}\right)}^{2}+{\left({\mathrm{PI}}_{\max}\right)}^{2}}{n}}$	n—the total number of elements, PI—the value of the single index, PI_max_—the maximum value of the PI [55].

Notes: The corresponding soil contamination classifications are given in Appendix A.

**Table 3:** Soil background values and toxicity factors for Jilin province were used in the calculations.

Elements	Soils Background Values (mg⋅kg^−1^)	Toxicity Factor
Cr	46.7	2
Cu	17.1	1
Zn	80.4	5
As	8.38	10
Pb	28.8	5

**Table 4:** Basic statistics of the concentrations measured by in situ pXRF, ICP-MS, and corrected pXRF in the urban area of Changchun City, China.

In Situ pXRF (mg⋅kg^−1^)	Mean	Max	Min
Cr	38.9	203.6	5.3
Cu	21.3	136.6	5.0
Pb	15.8	302.2	6.0
Zn	90.1	1511.6	27.8
As	11.2	21.8	4.0
ICP-MS (mg⋅kg^−1^)			
Cr	49.00	146.85	15.20
Cu	56.95	592.16	22.45
Pb	43.94	653.32	16.98
Zn	91.12	758.25	18.77
As	18.80	48.13	5.17
The corrected PXRF (mg⋅kg^−1^)			
Cr	64.22	167.68	38.93
Cu	43.78	392.10	3.90
Pb	57.43	565.72	13.44
Zn	96.23	795.20	39.66
As	20.90	39.86	6.40

**Table 5:** Correction equations and comparison of R-square before and after calibration.

Elements	Multiple Linear Regression Equations	R^2^ Before Correcting	R^2^ After Correcting	Degree of Advancement
Cr	Y = 0.652X + 0.001Ti + 0.954Si − 8.381	0.612	0.755	23.37%
Cu	Y = 1.196X + 0.009Ti − 15.030Si + 399.033	0.355	0.768	116.64%
Zn	Y = 0.493X − 0.017Ti − 9.932Si + 395.925	0.688	0.803	16.77%
As	Y = 1.785X + 0.001Ti − 0.569Si + 16.283	0.699	0.761	8.87%
Pb	Y = 1.795X − 0.008Ti − 4.254Si + 179.420	0.586	0.863	47.29%

Notes: where X denotes the content of pXRF of the element to be tested. Y represents the regression values of the element to be corrected. Silicon (Si) in units of 10^−2^ (%) and elemental titanium (Ti) in mg⋅kg^−1^.

**Table 6:** Statistical results of the comprehensive pollution index for the urban area of Changchun.

Index	Cr	Cu	Zn	As	Pb
PI-_Max_	3.59	22.93	9.89	5.02	19.64
PI-_avg_	1.38	2.56	1.20	2.68	1.99
CF	0.71	0.88	0.55	1.39	0.82
Nemerow index	1.20				

**Table 7:** Heavy metal element principal component analysis matrix and contribution of pollution sources.

Elements	Composition Matrix		Rotated Composition Matrix		Pollution Contribution		
	Component 1	Component 2	Component 1	Component 2	Source 1	Source 2	Unknown Source (s)
Cr	0.192	0.816	0.015	0.838	0.16%	69.37%	30.47%
Cu	0.916	0.081	0.878	0.273	30.89%	37.50%	31.61%
Zn	0.941	−0.253	0.974	−0.049	50.64%	13.17%	36.19%
As	0.257	0.809	0.081	0.845	2.30%	83.20%	14.50%
Pb	0.923	−0.217	0.948	−0.017	67.65%	8.26%	24.09%

**Table 8:** Environmental impact analysis based on the Leopold Matrix.

	Physiological Environment			Social Environment		Economic Environment	
Impact Activities	Soil	Water	Air	Infrastructure	Health	Employment	Value of Land
Raw materials for metal production	−2/3	−2/3	−1/3	−2/2	−1/3	1/3	−1/2
Vehicle manufacturing and assembly	−2/3	−2/2	−1/3	−1/2	−2/3	4/3	−1/2
Recycling and disposal of abandoned cars	−4/3	−2/2	−2/3	−1/2	−2/3	2/3	−2/2
Input of chemical raw materials	−3/2	−3/3	−1/3	-	−3/3	1/3	-
Product manufacturing	−4/3	−3/3	−2/3	−1/2	−3/3	4/3	−1/2
Treatment of waste biological products	−3/3	−2/3	−1/3	-	−3/3	3/3	−2/2
Fossil fuel usage	−3/3	−1/2	−4/3	−1/1	−3/3	1/2	-
Waste gas and waste residue emissions	−2/3	−3/3	−4/3	-	−3/3	1/2	−1/2
Fertilizer and pesticide usage	−1/2	−3/3	−1/3	−2/2	−3/3	-	−3/3
Vehicle exhaust emission	−2/2	−1/3	−2/3	-	−1/3	-	−1/3
Sewage disposal	−2/3	−3/3	−1/2	−1/2	−3/3	−1/2	-
Solid waste emissions	−3/3	−2/3	−1/2	−2/2	−3/3	−1/2	−1/3

## Data Availability

The data presented in this study are available on request from the corresponding author.

## Appendix A. Pollution Index Classification Scale

**Table:**

Index	Numerical Range	Classes
Single pollution index (PI)	PI < 1	non-polluting
	1−2	lightly polluted
	2−3	moderately polluted
	PI > 3	heavily polluting
Geo-accumulation index (I-geo)	I−geo < 0	non-polluted
	0−1	Uncontaminated to moderately contaminated
	1−2	Moderately contaminated
	2−3	Moderately to strongly contaminated
	3−4	Strongly contaminated
	4−5	Strongly to extremely contaminated
	I-geo > 5	Extremely high contaminated
Contamination Factor (CF)	CF < 1	non-polluting
	1−3	lightly polluted
	3−6	moderately polluted
	CF > 6	heavily polluted
Enrichment factor (EF)	EF < 2	Minimal enrichment
	EF = 2−5	Moderate enrichment
	EF = 5−20	Significant enrichment
	EF = 20−40	Very high enrichment
	EF > 40	Extremely high enrichment
The pollution load index (PLI)	PLI < 1	Clearly
	PLI > 1	Polluted
Risk factor (RI)	RI < 20	Low ecological risk
	20−40	Moderate ecological risk
	40−80	Considerable ecological risk
	80−160	High ecological risk
	>160	Serious ecological risk
Degree of contamination (C-_deg_)	C−_deg_ < 8	non-polluting
	8−16	lightly polluted
	16−32	moderately polluted
	C−_deg_ >32	heavily polluted
Nemerow pollution Index (PI-Nemerow)	PIN < 0.7	Class I soils (unpolluted)
	0.7−1	Class II soils (Safety)
	1−2	Class III soils (Mild pollution)
	2 −3	Super tertiary soils (Moderated)
	PIN > 3	Severe pollution
